# Supplementary figures and images for: A structured evaluation of genome-scale constraint-based modeling tools for microbial consortia
Source: PLoS Comput Biol. 2023 Aug 14;19(8):e1011363. doi: 10.1371/journal.pcbi.1011363 (PMC10449394; doi:10.1371/journal.pcbi.1011363)

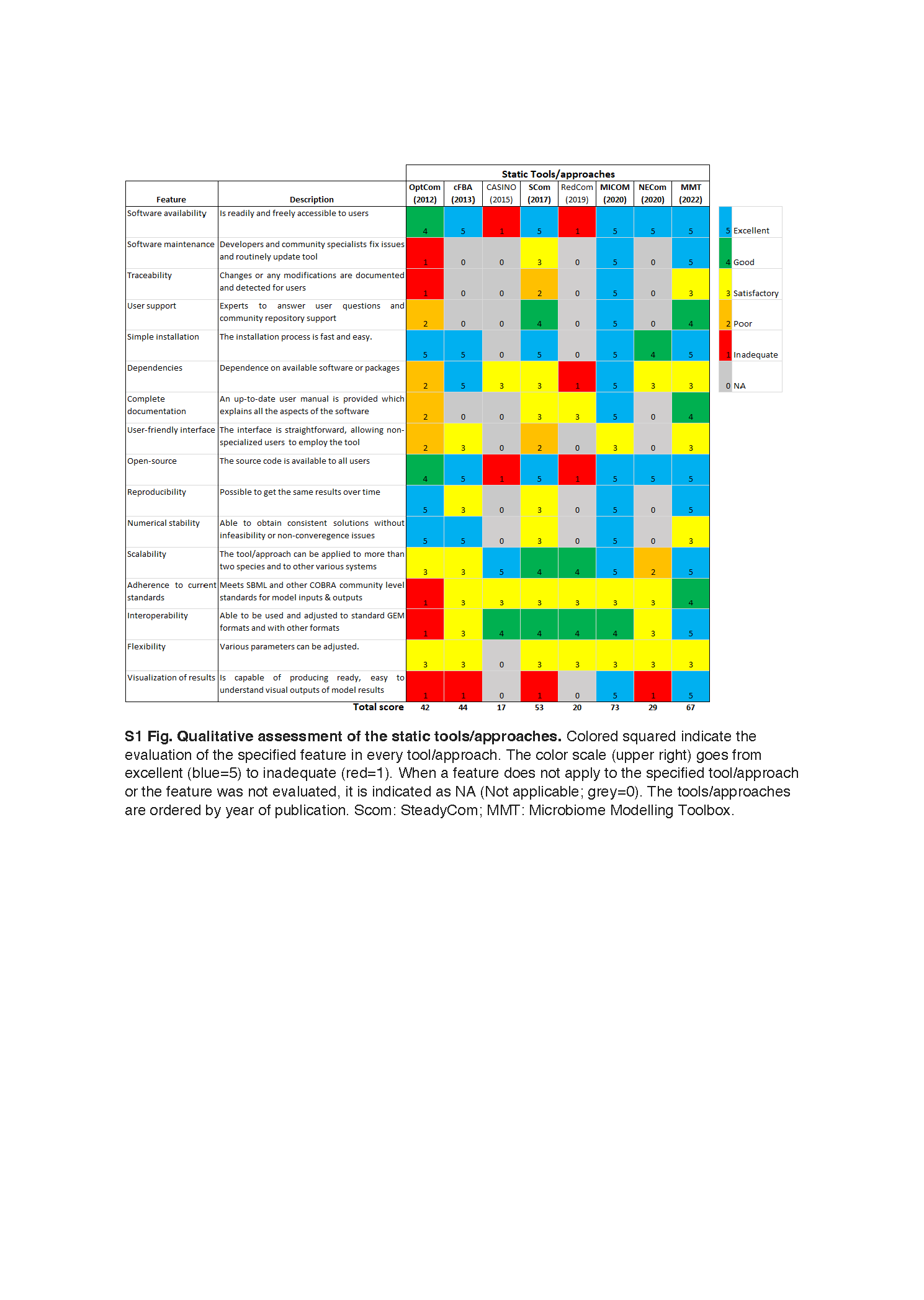

Supplement: S1 Fig — (TIF) [file pcbi.1011363.s001.tif]

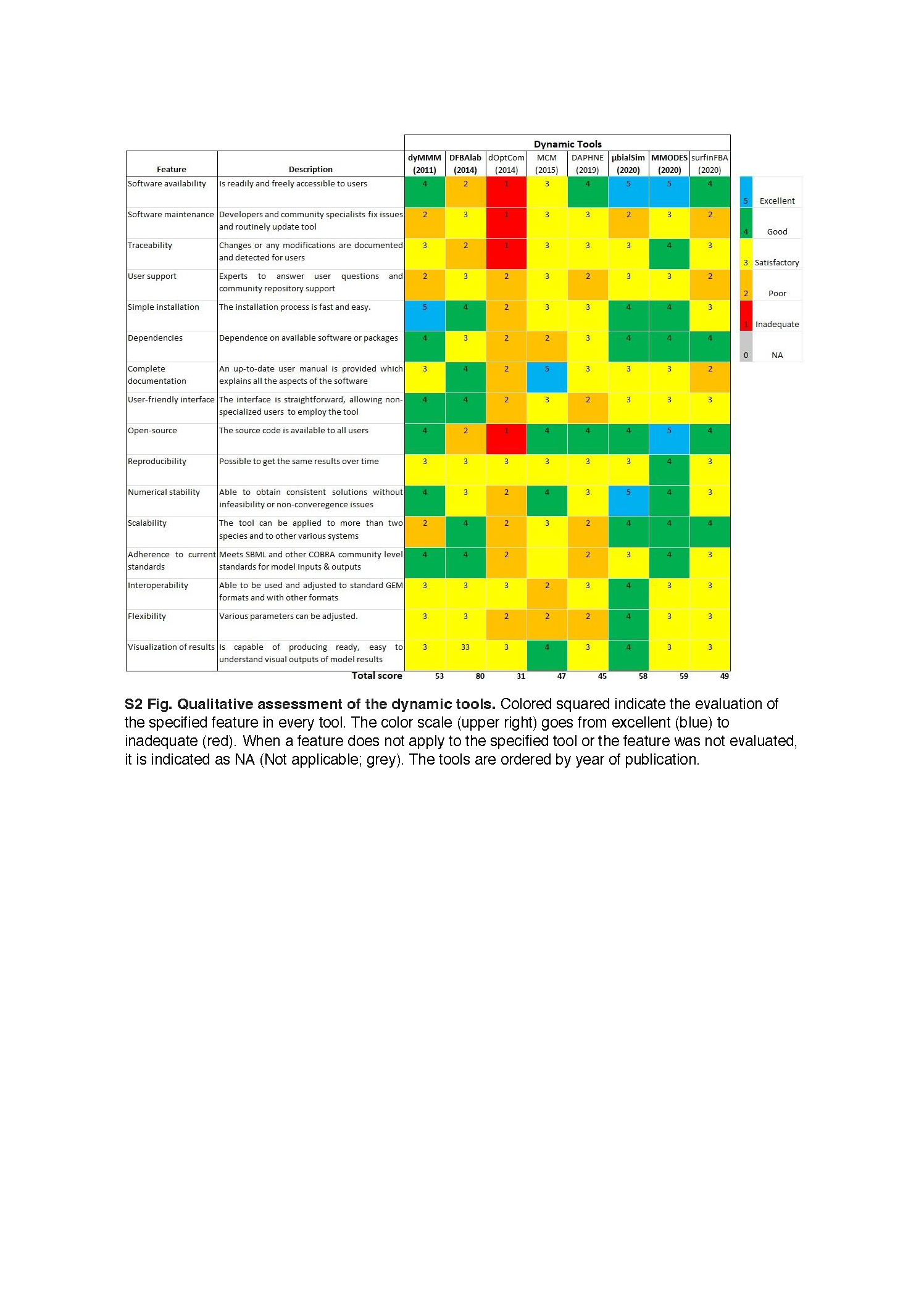

Supplement: S2 Fig — (TIF) [file pcbi.1011363.s002.tif]

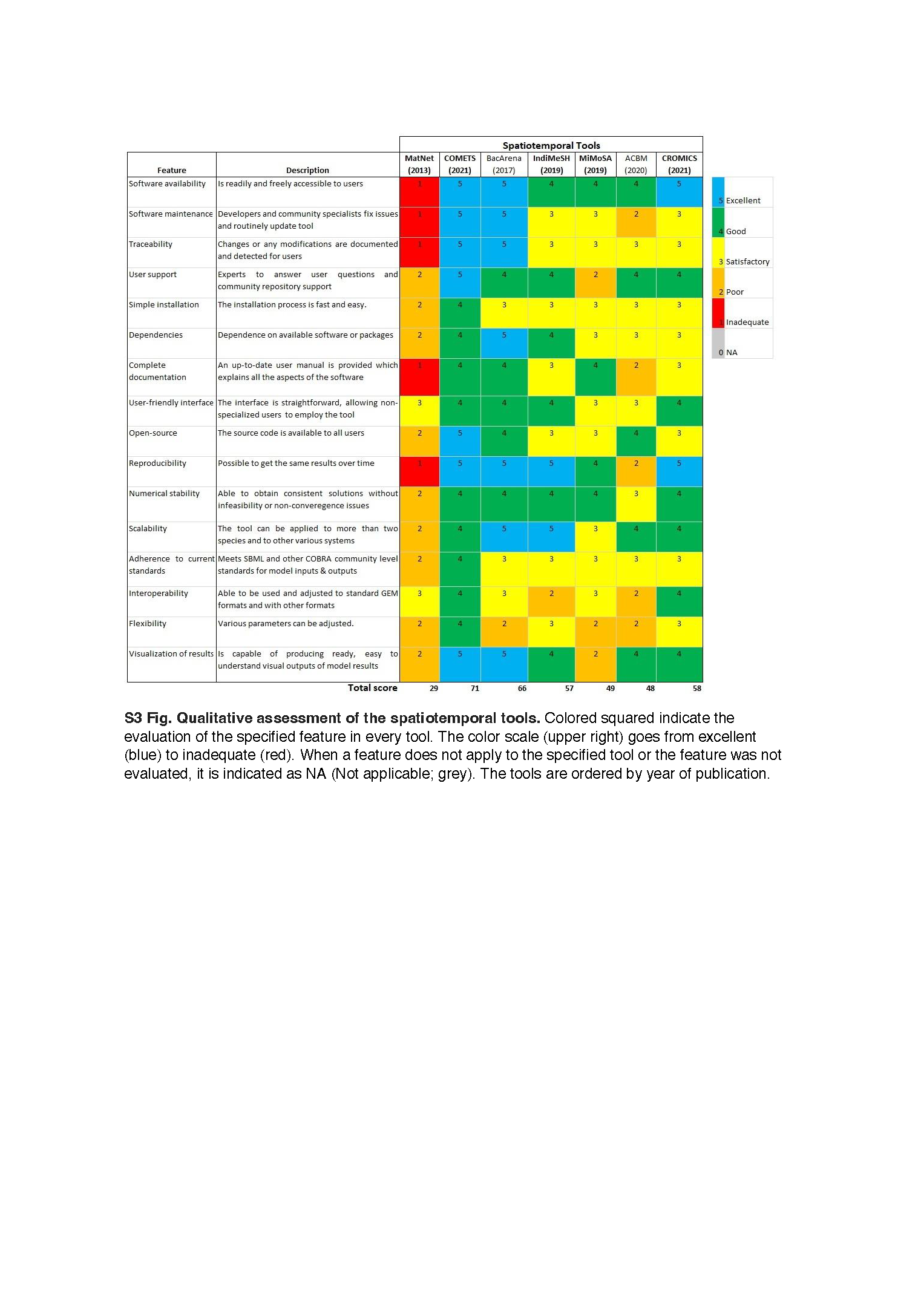

Supplement: S3 Fig — (TIF) [file pcbi.1011363.s003.tif]
