# Supplementary material for: A structured evaluation of genome-scale constraint-based modeling tools for microbial consortia
Source: PLoS Comput Biol. 2023 Aug 14;19(8):e1011363. doi: 10.1371/journal.pcbi.1011363 (PMC10449394; doi:10.1371/journal.pcbi.1011363)
Supplement: S1 Table — (PDF) [file pcbi.1011363.s004.pdf]

S1 Table. Summarized comparison of the available static tools/approaches.

Modeling Tool/approach (Year Developed)	Website/GitHub Link	Optimization Routine (Single or Bilevel)	Programming Language	Environment Dependencies	Optimization Dependencies	Namespace Requirement	# of citations (as of December 2022)
**OptCom (2012)**	http://www.maranasgroup.com/submission/OptCom.htm	Bilevel	GAMS[1]		BARON[2]	Yes	285
**cFBA (2013)**	https://journals.plos.org/plosone/article?id=10.1371/journal.pone.0064567	Single	Python	CBMPy	LP solver or CPLEX[3]	No	149
**CASINO (2015)**	Not Available	Bilevel	MATLAB[4]	RAVEN	No information	No	271
**SteadyCom (2017)**	https://github.com/opencobra/cobratoolbox/tree/master/src/analysis/multiSpecies/SteadyCom	Single	MATLAB[4]	COBRA Toolbox	LP solver or CPLEX[3]	No	133
**RedCom (2019)**	Not Available	Bilevel	MATLAB[4]	*CellNetAnalyzer*	CPLEX[3], *efmtool, fmincon*	No	24
**MICOM (2020)**	https://micom-dev.github.io/micom/	Single	Python	COBRApy	OSQP, CPLEX[3], Gurobi[5]	No	39
**MMT (v1, 2018; v2,2022)**	https://github.com/opencobra/cobratoolbox/blob/master/src/analysis/multiSpecies/microbiomeModelingToolbox/README.md	Single	MATLAB[4]	COBRA Toolbox	LP solver	No	79+3
**NECom (2020)**	https://github.com/Jingyi-Cai/NECom.git.	Bilevel	MATLAB[4]		BARON[2] and Gurobi[5], CPLEX[3] and glpk	No	12

[1] https://www.gams.com/latest/docs/UG_License.html
[2] https://www.minlp.com/baron-licenses
[3] https://www.ibm.com/products/ilog-cplex-optimization-studio/pricing
[4] https://nl.mathworks.com/pricing-licensing.html
[5]https://www.gurobi.com/academia/academic-program-and-licenses
