# Supplementary material for: A structured evaluation of genome-scale constraint-based modeling tools for microbial consortia
Source: PLoS Comput Biol. 2023 Aug 14;19(8):e1011363. doi: 10.1371/journal.pcbi.1011363 (PMC10449394; doi:10.1371/journal.pcbi.1011363)
Supplement: S2 Table — (PDF) [file pcbi.1011363.s005.pdf]

S2 Table. Rubric - Qualitative assessment of tools/approaches.

Features	Excellent=5	Good=4	Satisfactory=3	Poor=2	Inadequate=1
**Software availability**	Software is freely available for all users	Software is available upon user request	Access to the software tool is accessible for the majority of users	Access to the software tool is limitted to some users	Software is not available
**Software maintenance**	Tool is updated very regularly since publication. All issues are fixed in every version of the tool.	The majority of issues are fixed and tool is regularly updated	The tool has had some updates since the publication and some issues are fixed from time to time.	The tool rarely had an update or the last update registered was long time ago.	The tool has not undergone updated since publication.
**Traceability**	Changes are clearly documented and detected for users. Every version has information on the applied changes	The majority of changes and modifications are well detected and notified in every version for users.	Latest changes and modifications are documented and detected for users.	Changes are not clearly documented and detected for the user.	Changes are not documented and detected for the user
**User support**	The tool has several communication channels and user can easily get support from experts.	Experts answer questions routinely via a community repository support.	Experts answer questions from time to time via a community repository support.	There is no community repository support. Developers only provide a contact e-mail/person.	Developers don't provide a contact person nor a community support
**Simple installation**	Installation is not needed (tool available in COBRA Toolbox, COBRApy) or it is very easy and fast (e.g few pieces of code).	Installation is easy and fast	The installation process is relatively easy and moderately fast.	The installation process is slow and difficult.	The installation process is very slow and difficult for the user. The user requires help from the developers.
**Dependencies**	All the required packages and solvers are freely available for the user.	Most of required packages and solvers are free for a wide group of users.	Required software or packages are inexpensive for a wide group of users (e.g students, academics).	Required software or packages are not free and their use is not extended among users.	Required dependecies are not available.
**Complete documentation**	Up-to-date and extensive user manuals explaining all the aspects of the tool.	Moderately up-to-date and extensive user manuals explaining most of the aspects of the tool.	The tool has some tutorials available that integrate case studies covering some aspects of the tool. Rarely up to date.	The tool has few information available and does not include enough information to run the tool. It is not up-to-date	The tool does not provide any manual.
**User-friendly interface**	The tool is straightforward. All users with or without knowledge on programming and constrained-based modeling can run the tool.	Most of users with or without knowledge on programming and constrained-based modeling can run the tool.	The tool can be run by a user with some knowledge on programming, and contrained-based modeling.	The tool can be run by a user with extensive knowledge on programming, command-line, and contrained-based modeling .	A specialized user cannot run the tool with or without help from the community support.
**Open-source**	Code is freely available for all users	Code is available upon user request	Access to the tool code is accessible for the majority of users	Access to the tool code is limitted to some users	Code is not available
**Reproducibility**	The tool leads exactly to the same results over time	The results obtained over time are practically identical	Resutls obtained over time might be slightly different	Results obtained over time are different	Results are completely different over time.
**Numerical stability**	The tool always leads to consistent solutions without infeasibilities or non-convergence issues.	The tool leads to consistent solutions almost without infeasibilities or non-convergence issues.	The tool leads to inconsistent solutions with infeasibilities or non-convergence issues in few cases.	The tool leads to inconsistent solutions with infeasibilities or non-convergence issues in many cases.	The tool does not lead to consistent solutions.
**Scalability**	The tool can be applied to very large communities (>100) and various systems.	The tool can be applied to large communities (~ up tp 10) and various systems.	The tool can be applied to model medium communities (~up to 3-4 species).	The tool is only applied to model small communities (~up to 2 species)	The tool cannot be applied to model small communities
**Adherence to current standards**	The tool meets SBML and COBRA community level standards for model inputs and outputs.	The tool meets SBML and COBRA community standards for model inputs and SBML or COBRA community standards for outputs.	The tool meets SBML or COBRA community level standards for model inputs & outputs files.	Only the input or the output file meet SBML or other COBRA community level standards.	Input and output files don't meet any community level standard.
**Interoperability**	The tool can be used and adjusted to several GEM formats with and without using an external package.	The tool can be used and adjusted to several GEM formats using external packages (e.g COBRA Toolbox, COBRApy)	The tool can be used and adjusted to one format.	The tool can be used with one format but not be adjusted.	The tool cannot be used and adjusted to standard GEM and with other formats.
**Flexibility**	Many parameters can be adjusted in an easy way.	Some parameters can be adjusted relatively easy.	Some parameters can be adjusted easily and some others less easy.	Very few parameters can be adjusted and there are many difficulties.	Any parameter can be adjusted.
**Visualization of results**	The tool produces many ready and easy to understand visual outputs of model results.	The tool mostly produces ready and easy to understand visual outputs of model results.	The tool produces some visual outputs that are almost ready and relatively easy to understand.	The tool provides few visual outputs that need to be adjusted.	The tools does not produce visual outputs.
