# Supplementary material for: A structured evaluation of genome-scale constraint-based modeling tools for microbial consortia
Source: PLoS Comput Biol. 2023 Aug 14;19(8):e1011363. doi: 10.1371/journal.pcbi.1011363 (PMC10449394; doi:10.1371/journal.pcbi.1011363)
Supplement: S3 Table — (PDF) [file pcbi.1011363.s006.pdf]

S3 Table. **Inputs, outputs, and assumptions of the static tools/approaches.**

Tool/approach	Inputs	Outputs	Assumptions
**OptCom**	GEM of single species, CO uptake rate, relative abundance, min. growth rate of species, total biomass.	Community growth rate, species growth rate, fluxes	Steady-state, species don't grow at balanced growth.
**cFBA**	Community GEM, CO uptake rate, relative abundance, total biomass, species growth rate.	Community growth rate, fluxes	Steady-state, equal growth rates of species and community.
**SteadyCom**	GEM of single species, CO uptake rate, total biomass.	Relative abundance, Community growth rate, species growth rate, fluxes	Steady-state, equal growth rates of species and community.
**MMT**	GEM of single species, CO uptake rate, relative abundance, total biomass, species growth rate.	Community growth rate, fluxes	Steady-state, equal growth rates of species and community.
**MICOM**	GEM of single species, CO uptake rate, relative abundance, total biomass.	Community growth rate, species growth rate, fluxes	Steady-state, species don't grow at balanced growth.
