# Supplementary material for: A structured evaluation of genome-scale constraint-based modeling tools for microbial consortia
Source: PLoS Comput Biol. 2023 Aug 14;19(8):e1011363. doi: 10.1371/journal.pcbi.1011363 (PMC10449394; doi:10.1371/journal.pcbi.1011363)
Supplement: S4 Table — (PDF) [file pcbi.1011363.s007.pdf]

S4 Table. **Genome-scale metabolic models (GEMs) and input parameters used as constraints in some static tools/approaches to model the co-culture of *C. autoethanogenum* and *C. kluyveri*.** These values shown are used only when the specific parameter was considered as an input parameter of that specific tool/approach (see S2 Table).

Parameter	*C. autoethanogenum*	C. kluyveri	Community
**GEM**	iCLAU786 [67]	ickl708 [68]	Multi-species GEM [54]
**CO uptake rate (mmol L^{-1} h^{-1})**			4.8 [53]
**Relative abundance**	0.4 [54]	0.6 [54]	
**Total biomass (g)**			0.22 [53]
**Growth rate (h^{-1})**	0.021 [53] min. 0.005	0.021 [53] min. 0.005	
