# Supplementary material for: A structured evaluation of genome-scale constraint-based modeling tools for microbial consortia
Source: PLoS Comput Biol. 2023 Aug 14;19(8):e1011363. doi: 10.1371/journal.pcbi.1011363 (PMC10449394; doi:10.1371/journal.pcbi.1011363)
Supplement: S5 Table — (PDF) [file pcbi.1011363.s008.pdf]

**S5 Table. Summarized comparison of the available dynamic tools/approaches.**

| Modeling Tool (Year Developed) | Website/GitHub Link | Optimization Routine (Single or Bilevel) | Programming Language | Environment Dependencies | Optimization Dependencies | Namespace Requirements | # of citations (as of December 2022) | Potential Software Licenses Needed |
|---|---|---|---|---|---|---|---|---|
| **DyMMM (2011)** | https://sourceforge.net/p/dymmm/wiki/Home/ | Single | MATLAB[1] | COBRA Toolbox | CPLEX[2], Mosek[3], or Gurobi[4] | No | 206 | |
| **DFBAlab (2014)** | https://yoric.mit.edu/software/dfbalab/how-obtain-dfbalab#gsc.tab=0 | Single | MATLAB[1] | COBRA Toolbox | CPLEX[2], Mosek[3], or Gurobi[4] | No | 83 | |
| **MMODES (2019)** | https://mmodes.readthedocs.io/en/latest/ | Bilevel | Python | COBRApy | GLPK | BiGG | 2 | |
| ***μ*BialSim (2020)** | https://www.frontiersin.org/articles/10.3389/fbioe.2020.00574/full | Single | MATLAB[1] | CobraToolbox/CellNetAnalyzer | GLPK | No | 27 | |

[1] https://nl.mathworks.com/pricing-licensing.html

[2] https://www.mosek.com/products/academic-licenses/

[3] https://www.ibm.com/products/ilog-cplex-optimization-studio/pricing

[4] https://www.gurobi.com/academia/academic-program-and-licenses
