# Supplementary material for: A structured evaluation of genome-scale constraint-based modeling tools for microbial consortia
Source: PLoS Comput Biol. 2023 Aug 14;19(8):e1011363. doi: 10.1371/journal.pcbi.1011363 (PMC10449394; doi:10.1371/journal.pcbi.1011363)
Supplement: S6 Table — (PDF) [file pcbi.1011363.s009.pdf]

S6 Table. **Inputs, outputs, and assumptions of the dynamic tools/approaches.**

Tool/approach	Inputs	Outputs	Assumptions
**DyMMM**	Genome-scale metabolic models; Initial volume, biomass, and substrate concentrations; duration (time span) of the experimental run; exchange metabolites (reactions) of interest, Michaelis-Menten kinetics	Biomass, substate, and product formation over time, as well as metabolic fluxes at each time step	Reaction rates are constant over time intervals
**DFBAlab**	Genome-scale metabolic models; Initial volume, biomass, and substrate concentrations; duration (time span) of the experimental run; exchange metabolites (reactions) of interest, Michaelis-Menten kinetics	Biomass, substate, and product formation over time as well as metabolic fluxes at each time step	Reaction rates are constant over time intervals
**MMODES**	Genome-scale metabolic models; Initial volume, biomass, and substrate concentrations; duration (time span) of the experimental run; exchange metabolites (reactions) of interest, Michaelis-Menten kinetics	Biomass, substate, and product formation over time as well as metabolic fluxes at each time step	Reaction rates are constant over time intervals
***μ*BialSim**	Genome-scale metabolic models; Initial volume, biomass, and substrate concentrations; duration (time span) of the experimental run; exchange metabolites (reactions) of interest, Michaelis-Menten kinetics	Two files are generated at the end of the simulation with a date and time stamp in the filename indicating the start of the simulation. The field's time, compounds, biomass, and mu hold the time, compound concentrations, biomass concentrations, and specific growth rates for each integration step. The field FBA stores data for each FBA model, including the temporal dynamics of all metabolic fluxes, and the mass balance for all exchange reactions.	Reaction rates are constant over time intervals.
