# Supplementary material for: A structured evaluation of genome-scale constraint-based modeling tools for microbial consortia
Source: PLoS Comput Biol. 2023 Aug 14;19(8):e1011363. doi: 10.1371/journal.pcbi.1011363 (PMC10449394; doi:10.1371/journal.pcbi.1011363)
Supplement: S7 Table — (PDF) [file pcbi.1011363.s010.pdf]

S7 Table. Genome-scale metabolic models (GEMs), GEM constraints, substrate uptake parameters, and initial biomass concentrations used by some dynamic tools/approaches to model the co-culture of *S. cerevisiae* and *E. coli* These values shown are used only when the specific parameter was considered as an input parameter of that specific tool/approach (see S2 Table).

Parameter	*E. coli*	*S. cerevisiae*
**GEM**	iJR904	iND750
**GEM Modifications**	Glucose Kinase, GLUK, LB=UB=0<br>Glucose Exchange, EX_glc(e), LB=UB=0	-
**vgmax (mmol/gDW/h)**	-	25.9
**Kg (g/L)**	-	0.5
**vz,max (mmol/gDW/h)**	9	-
**Kz (g/L)**	0.01	-
**Ki,e (g/L)**	8	10
**vo,max (mmol/gDW/h)**	8	1.5
**Ko (mmol/L)**	0.001	0.003
**Initial Biomass (gDW/L)**	0.05	0.05
