# Supplementary material for: A structured evaluation of genome-scale constraint-based modeling tools for microbial consortia
Source: PLoS Comput Biol. 2023 Aug 14;19(8):e1011363. doi: 10.1371/journal.pcbi.1011363 (PMC10449394; doi:10.1371/journal.pcbi.1011363)
Supplement: S8 Table — (PDF) [file pcbi.1011363.s011.pdf]

S8 Table. Initial substrate concentrations used for the co-culture dynamic tools/approaches model the co-culture of *S. cerevisiae* and *E. coli*.

Metabolite	Initial Concentration (mmol/L)
Glucose	88.80
Xylose	53.28
Ethanol	0
Oxygen	0.24
