# Supplementary material for: A structured evaluation of genome-scale constraint-based modeling tools for microbial consortia
Source: PLoS Comput Biol. 2023 Aug 14;19(8):e1011363. doi: 10.1371/journal.pcbi.1011363 (PMC10449394; doi:10.1371/journal.pcbi.1011363)
Supplement: S9 Table — (PDF) [file pcbi.1011363.s012.pdf]

S9 Table. Summarized comparison of the available spatiotemporal tools/approaches.

Modeling Tool (Year Developed)	Website/GitHub Link	Optimization Routine (Single or Bilevel)	Programming Language	Environment Dependencies	Optimization Dependencies	Namespace Requirements	# of citations (as of December 2022)	Potential Software Licenses Needed
**COMETS (2014, 2021)**	https://www.runcomets.org	Single	MATLAB[1], Python, Command-line	COBRA Toolbox, COBRAPy	CPLEX[2], Mosek[3], or Gurobi[4]	BiGG	312	
**BacArena (2017)**	https://bacarena.github.io	Single	R	Sybil	CPLEX[2], Mosek[3], or Gurobi[4]	No	144	
**IndiMesh (2019)**	https://journals.plos.org/ploscompbiol/article?id=10.1371/journal.pcbi.1007127	Bilevel	MATLAB[1]	-	GLPK	No	32	
**CROMICS (2021)**	https://github.com/EPFL-LCSB/cromics	Single	MATLAB[1]	-	GLPK	No	3	

[1] https://nl.mathworks.com/pricing-licensing.html
[2] https://www.mosek.com/products/academic-licenses/
[2] https://www.ibm.com/products/ilog-cplex-optimization-studio/pricing
[3] https://www.mosek.com/products/academic-licenses/
[4]https://www.gurobi.com/academia/academic-program-and-licenses
