# Supplementary material for: A structured evaluation of genome-scale constraint-based modeling tools for microbial consortia
Source: PLoS Comput Biol. 2023 Aug 14;19(8):e1011363. doi: 10.1371/journal.pcbi.1011363 (PMC10449394; doi:10.1371/journal.pcbi.1011363)
Supplement: S10 Table — (PDF) [file pcbi.1011363.s013.pdf]

S10 Table. Inputs, outputs, and assumptions of the spatiotemporal tools/approaches.

Tool/approach	Inputs	Outputs	Assumptions
**COMETS**	Genome-scale metabolic models; Initial volume, biomass, and substrate concentrations; duration (time span) of experimental run; exchange metabolites (reactions) of interest, Michaelis-Menten kinetics, Diffusion parameters, Grid size	Biomass, substate, and product formation over time as well as metabolic fluxes at each time step, spatial locations of microbial species	Reaction rates are constant over time intervals. Metabolite diffusion coefficient is constant
**BacArena**	Genome-scale metabolic models; Initial volume, biomass, and substrate concentrations; duration (time span) of experimental run; exchange metabolites (reactions) of interest, Michaelis-Menten kinetics, Diffusion parameters, Grid size	Biomass, substate, and product formation over time as well as metabolic fluxes at each time step, spatial locations of microbial species	Reaction rates are constant over time intervals. Metabolite diffusion coefficient is constant.
**IndiMesh**	Genome-scale metabolic models; Initial volume, biomass, and substrate concentrations; duration (time span) of experimental run; exchange metabolites (reactions) of interest, Michaelis-Menten kinetics, Diffusion parameters, Pore size	Biomass, substate, and product formation over time as well as metabolic fluxes at each time step, spatial locations of microbial species	Reaction rates are constant over time intervals. Metabolite diffusion coefficient is constant
**CROMICS**	Genome-scale metabolic models; Initial volume, biomass, and substrate concentrations; duration (time span) of experimental run; exchange metabolites (reactions) of interest, Michaelis-Menten kinetics, Diffusion parameters, Box size, the volume occupied by cells	Biomass, substate, and product formation over time as well as metabolic fluxes at each time step, spatial locations of microbial species, crowding conditions of the cells	Reaction rates are constant over time intervals.
