# Supplementary material for: A structured evaluation of genome-scale constraint-based modeling tools for microbial consortia
Source: PLoS Comput Biol. 2023 Aug 14;19(8):e1011363. doi: 10.1371/journal.pcbi.1011363 (PMC10449394; doi:10.1371/journal.pcbi.1011363)
Supplement: S11 Table — (PDF) [file pcbi.1011363.s014.pdf]

S11 Table. Genome-scale metabolic models (GEMs) and input parameters used as constraints in some spatiotemporal tools/approaches to model the co-culture of *S. enterica* and *E. coli*. These values shown are used only when the specific parameter was considered as an input parameter of that specific tool/approach (see S2 Table).

Parameter	*E. coli*	*S. enterica*
**GEM**	iJO1366	iiRR1083
**GEM Modifications**	Cystathionine γ-synthase)., *metB*, LB=UB=0	gain-of-function mutations in metA (homoserine transsuccinylase) biomass = S0.reactions.BIOMASS_iRR1083_metals.add_metabolites({met_c : -.5, met_e : .5}) -
**Vmax (mmol/gDW/h)**	10 -	10
**Km (μM)**	10	10
**Death rate**	1%	1%
**Metabolite diffusion (cm2/s)**	0.01	0.01
**Biomass diffusion (cm^2/s)**	8	8
**Max. colony height (200 μm)**	8	8
**Oxygen concentration (μmol/cm^2)**	250	250
