# Supplementary material for: A structured evaluation of genome-scale constraint-based modeling tools for microbial consortia
Source: PLoS Comput Biol. 2023 Aug 14;19(8):e1011363. doi: 10.1371/journal.pcbi.1011363 (PMC10449394; doi:10.1371/journal.pcbi.1011363)
Supplement: Attachment — Submitted filename: PLOSComp-Rev1-ResponseLetter_1_Submission.docx [file pcbi.1011363.s017.docx]

**Ms. Ref. No.:	PCOMPBIOL-D-23-00221-****PLOS Computational Biology**

**Manuscript Title:** A Structured Evaluation of Genome-Scale Constraint-Based Modeling Tools for Microbial Consortia

**Article Type:** Original research paper

Dear Prof. Tunahan Çakır, Ph.D. – Guest Editor of *PLOS Computational Biology* and Prof. Kiran Patil, Ph.D. – Section Editor of *PLOS Computational Biology*,

We would like to thank the reviewers for their insightful comments. Our responses to their comments are listed below (comments and questions from a reviewer are in bold, followed by our responses). The lines indicated in the comments correspond to the new version of the manuscript. We believe we have improved the manuscript with the reviewer’s constructive comments and hope it is ready for publication.

******Reviewer 1******

# GENERAL COMMENT 1

# Scott Jr et all present a timely and complete review addressing the comparative analysis of available software for microbial communities modelling. The authors address the detailed analysis of steady-state, dynamic and spatio-temporal modelling tools. The authors rank the tools according their performance but also in the context of FAIR principles. The dataset of selected tools is very complete and very representative of the state of the field. There are some weaknesses limiting the scope of the review, I suggest to address some of them in order to increase the value of the current manuscript.

We feel pleased that your description recognizes the detail and effort we invested into the performed modeling analysis. Also, we appreciate you acknowledging the utility of our work and pointing out certain parts of the manuscript that can be improved after revision. The following contains responses to the specific comments:

**MAJOR COMMENTS**

**Below the authors will find some comments that will help to improve the article**

**1. The review addresses the analysis of steady-state, dynamic, and spatiotemporal tools, however, there are interesting alternatives to analyse microbial communities such as SMETANA (Zelezniak et al 2014) and** **Community Gap-filling (Giannari et al 2021) which** **identify interspecific interactions or** **DOLMN (Thommes et al 2019) and FLYCOP (Garcia-Jimenez et al 2018) which address the synthetic designing of microbial consortia. In order to increase the scope of the review perhaps such methods could be reviewed and ranked as well.**

We appreciate this recommendation to improve the scope of the manuscript. However, we feel that many of the tools the reviewer suggested adding to the study are outside of the scope of this work. This review, on the contrary, evaluates GEM modeling tools for microbial communities where the user needs to provide draft or curated GEMs as inputs. A more suitable review, including tools such as Community Gap-Filling, was published by Mendoza and coworkers in 2019 [1], where they surveyed genome-scale metabolic reconstruction software platforms. For example, while the Community Gap-Filling [2] tool is capable of identifying interspecies relationships, it is a tool devised primarily to resolve metabolic gaps in genome-scale metabolic reconstructions (GEM) at the community level.

We elected to exclude tools such as DOLMN and FLYCOP from this study’s assessments because we believe tools such as these pertain to a subset of approaches, are fully integrated pipelines that rely on reviewed tools, or are ancillary. For instance, the DOLMN tool is applicable for investigating the division of labor or interactions among different strains within the same species. Moreover, the FLYCOP pipeline employs the COMETS version 1 tool, which is already included in this study’s assessment. Therefore, we feel that FLYCOP is more appropriate as an auxiliary tool to facilitate the *in-silico* design of synthetic microbial communities. We acknowledge that the scope of tools covered in this study may be unclear to the reader. As a result, we have added a more thorough explanation of the scope of our study and mentioned the existence of these additional approaches in the Discussion section

as follows:

“In general, GEM modeling tools fall into several categories for analyzing microbial communities. They have been used to identify interspecific interactions, which are embodied in tools such as SMETANA [3]. These tools can also contain extensive functionality, as illustrated in the Community Gap-Filling [2] tool for creating draft GEMs from genome assemblies and performing gap filling and, based on the resolved metabolic gaps, can determine interspecies relationships. There are tools available such as CommModelPy [4], DOLMN [5], FLYCOP [6], and Community Opt-yield-FBA [7], which were developed to facilitate the synthetic designing of microbial consortia. However, this study focuses on static, dynamic, and spatiotemporal Flux-Balance-Analysis-based modeling tools used for simulating extracellular and intracellular metabolic phenotypes of microbial consortia. Furthermore, the scope of the work was limited to evaluating GEM modeling tools for microbial communities where the user needs to provide draft or curated GEMs as inputs and not tools that are used primarily to create the reconstructions. CommModelPy [4], DOLMN [5], and FLYCOP [6] likewise were omitted from this study’s assessment because we believe they are outside its scope. For instance, the DOLMN tool is applicable for investigating the division of labor or interactions among different strains within the same species. Moreover, the FLYCOP pipeline employs the COMETS version 1 tool, which is already included in this study’s assessment. Therefore, we feel that FLYCOP is more appropriate as an expansion tool to facilitate the *in-silico* design of synthetic microbial communities.”

Please see lines: 489 -509

**2. To increase the understanding of the review I suggest to include potential software licenses associated with each method since it is an important aspect when selecting the software.**

Thanks for suggesting. We have included a link to the potential software licenses associated with each method in the supplementary Table S1, Table S5, and Table S9.

To reflect on this point the following has been added to the manuscript “Licenses are also important and should be provided because they allow tools to be incorporated in pipelines such as FLYCOP that build upon and extend the functionalities of COMETS to design microbial communities” For these additions, see lines 557-559.

**3.** **In the case of the assignment of flexibility is required a more detailed information from the authors. For me it’s not clear what mean satisfactory or good, for instance. Overall, I miss a more detailed description of the qualitative measures used to create the different scores used in the text. They look actually very subjective. There are multiple qualitative measures, but some of the variables can be objectively measured. For instance, the reproducibility or scalability of the data can be quantitatively analysed against available data to corroborate the score.**

We agree that adding more explanation regarding the metrics used in the qualitative assessment will help the reader interpret our results more resolutely. While the evaluation of some features can be subjective, we believe that the creation of an evaluation rubric helped to evaluate the tools in a consistent and fair way.

We have first clarified this fact and emphasized the existing evaluation rubric (presented in the supplementary material) created for the evaluation of these tools by adding the following lines:

Lines 130-132

"Some features can be subjective. Thus, a description of the criteria followed to evaluate each qualitative metric was described in an evaluation rubric (Supplementary File; S2 Table)”.

Lines 286-287:

“Fig.4 shows the evaluation of the described features in every tool/approach based on the evaluation rubric (S2 Table).”

Lines 382-383:

“Fig.7 shows the evaluation of the described features in every tool/approach based on the evaluation rubric (S2 Table).”

We realize that the names of the two features Scalability and Numerical Stability, were mistakenly switched. Therefore, we have switched the description of scalability and numerical stability in Fig 2, 4, 7, S1, S2 and S3. Now, “Scalability” is referring to: "The tool can be applied to more than two or to various systems.” “Numerical Stability” refers to: “Able to obtain consistent solutions without infeasibility or non-convergence issues.”

We recognized this was not clear in the manuscript and therefore, we have included the following lines:

“More specifically, to evaluate the numerical stability and reproducibility of the tools, we relied on existing literature, tool architecture and/or their performance in reproducing the available experimental studies chosen for the quantitative assessment. Not all features could be examined since some tools were not readily available, there was no tool developed for those approaches or there was a lack of related information in literature. These features were marked as ‘Not applicable' (NA; grey square).”

Please see lines: 132 -138

Furthermore, we added an additional statement in the Discussion section that underscores our reasoning and the need for more suitable data to be publicly available. The following statement has been added: “By having more publicly available datasets, GEM modeling tools would be able to be more robustly evaluated on a quantitative level in terms of their scalability and numerical stability.”

Please see lines: 529-531.

**4. Overall, I miss some software traceability in term of software version. The authors don’t mention the software versions used, or downloading date. This is important for the reproducibility of the analysis.**

We agree, and we have added a statement that “all tools evaluated in this study are the most up-to-date versions as of December 15, 2022.” See lines 654-655 for the added statement. Furthermore, the specific tool version has been added in the Materials and Methods section of every tool, and the scripts included in the online repositories: <https://doi.org/10.5281/zenodo.7573135> and <https://gitlab.com/wurssb/Modelling/modelingtools_microbial_consortia>

**5.** **In discussion, the authors observed that the qualitative assessment correlate to the quantitative assessment, but they tested only the tools with highest qualitative score, so this assumption is somewhat weak. Can the authors extend the discussion of this topic?**

We thank the reviewer for this observation. We have added the following in the discussion to correct this point:

Lines 514-518:

“Tools that were less 'FAIR' could hardly be accessed nor used in this study. Thus, further information and access to the code would be required to quantitatively assess the performance of these tools and draw a conclusion. Nevertheless, a tool needs to be FAIR to be used, to contribute to the field, and thus, to shed light on the continuous challenges that can be, later on, tackled in new tools.”

**6. The figures in general have low resolution, and the text and numbers of the axis are too small.**

We agree and thank the reviewer for pointing out these issues with the quality of the figures. We have corrected these by including higher-resolution figures and enlarging the font size within the figures.

**7. The authors omit the version/year of publication of the tools in the figures 2,4 and 7. So the potential readers cannot identify the version of the software used. I suggest adding this information in the legend.**

Fig 2, 4, and 7 already show the name of the tool and below the year of the latest publication. Every legend specifies that tools are ordered by the year of the latest publication.

We have included the version of every tool in the Materials and Methods section and the corresponding scripts as referenced in comment 4.

**8. I miss the scripts used by the authors for the analysis following FAIR principles.**

The code used to produce our reported results can be downloaded from: <https://doi.org/10.5281/zenodo.7573135> and [https://gitlab.com/wurssb/Modelling/modelingtools_microbial_consortia](https://gitlab.com/wurssb/Modelling/modelingtools\_microbial\_consortia). Besides the parts where the links are given in the main text, we have added a section called "Supporting information,” where we have included the same links. The second link correspond to a new GitLab repository where we have included the same scripts that are in the zenodo repository and README files with detailed information to run each type of tools.

**MINOR COMMENTS**

**1. Line 398 COBRAPy to COBRApy**

Thanks for catching this. The syntax error has been corrected.

**2. How Agora 2.0 paper has been already published (https://www.nature.com/articles/s41587-022-01628-0 ) please use the new citation instead of the BioRxiv preprint.**

Thanks for pointing this out. We have now updated the manuscript to include a reference to the published Agora 2.0 paper found in Nature Biotechnology rather than the BioRxiv preprint version.

******Reviewer 2******

# GENERAL COMMENT 1

# I have enjoyed reading the manuscript entitled "A Structured Evaluation of Genome-Scale Constraint-Based Modeling Tools for Microbial Consortia" and found it to be a valuable and timely contribution to the field. The authors have done an excellent job in systematically evaluating COBRA-based tools for microbial communities, which addresses an important gap in the literature. This work is expected to provide the scientific community with guidance on selecting appropriate tools for their specific applications and to help developers prioritise improvements in the future.

# The manuscript is well-written and well-organised, with clear objectives and a comprehensive methodology. The qualitative assessment of the 24 published tools using the FAIR principles is informative, and the quantitative testing of a subset of 14 tools against experimental data from three different case studies is commendable. The authors' discussion on the differences in the mathematical formulation of the approaches and their relation to the results provides valuable insights and shows a deep understanding of the topic.

# However, I would like to suggest an improvement that could further enhance the rigour of the quantitative assessment. The authors could consider incorporating additional quantitative Key Performance Indicators (KPIs), such as error and correlation of dynamic behaviours of the metabolic system. These KPIs can help to evaluate the accuracy of the predictions made by the COBRA-based tools, and allow for a more comprehensive comparison between them. By including these metrics, readers will have a better understanding of the strengths and limitations of each tool and be better equipped to make informed decisions on which tool to use for their specific research question. Also in discussion one could add that every tool can be as accurate as the model is and that the reader should pay take all quantitive evaluations with a pinch of salt... And most important, besides just providing a link to zenodo repository, I could not find a documentation notebook that puts all results together or explain how to install entire set of analyses, this is absolutely crucial for publication.

# In conclusion, I believe that the manuscript is an important and well-executed study that will significantly contribute to the field of microbial community research. I recommend its acceptance for publication, with the suggested improvement to the quantitative assessment. This work will undoubtedly serve as a valuable resource for researchers and developers working with COBRA-based tools and microbial communities.

We feel pleased that your description recognizes the significance and content of our work. Also, we appreciate your concern regarding the quantitative Key Performance Indicators used in the study and the documentation not highlighted throughout our manuscript to emphasize how to perform the analyses. Therefore, we have augmented, modified, and reorganized substantial parts of the Methods, Results, and Discussion sections corresponding with the reviewer’s request to help underline the methods used in this work.

To tackle these comments specifically, we have added the following:

Lines 531-543:

“In general, the performance of these tools would be enhanced if the quality of the GEMs or model architecture was improved, and more constraints were applied to fit the experimental studies or to compute fluxes. However, we ran the tools without augmenting them and following the reported standards used to avoid an unbiased evaluation. The quantitative evaluations should be taken with a degree of awareness about their generality and projection to other types of systems. Furthermore, the quantitative evaluations here performed should be carefully considered as indications of tool performance rather than absolute measures of performance. They represent the behavior of the tools when confronted with specific datasets with defined biological characteristics. The syntrophic relationship established in the system used to test the static tools is different from the alleviation of toxicity observed in the dataset used for the dynamic tools. We cannot rule out that tool performance is affected by the nature of the interactions.”

We have created a new GitLab repository (see comments 4 and 8 from reviewer 1) with all the code used to run these tools including README files to guide the users. Each script is well documented and contains information on the software version used to run the tools. Furthermore, we have added all the software versions used to run the tools in the Material and Methods section.

Lines 658-662:

“The code used to produce our reported results can be downloaded from: <https://doi.org/10.5281/zenodo.7573135> and <https://gitlab.com/wurssb/Modelling/modelingtools_microbial_consortia>. The scripts can be used by other users as a guide to facilitate the evaluation and use of these tools.”

Sincerely,

Dr. William T. Scott, Jr.

**REFERENCES**

1.	Mendoza SN, Olivier BG, Molenaar D, Teusink B. A systematic assessment of current genome-scale metabolic reconstruction tools. Genome Biology. 2019;20(1):158. doi: 10.1186/s13059-019-1769-1.

2.	Giannari D, Ho CH, Mahadevan R. A gap-filling algorithm for prediction of metabolic interactions in microbial communities. PLOS Computational Biology. 2021;17(11):e1009060. doi: 10.1371/journal.pcbi.1009060.

3.	Zelezniak A, Andrejev S, Ponomarova O, Mende DR, Bork P, Patil KR. Metabolic dependencies drive species co-occurrence in diverse microbial communities. Proceedings of the National Academy of Sciences. 2015;112(20):6449-54. doi: doi:10.1073/pnas.1421834112.

4.	Bekiaris PS, Klamt S. Designing microbial communities to maximize the thermodynamic driving force for the production of chemicals. PLOS Computational Biology. 2021;17(6):e1009093. doi: 10.1371/journal.pcbi.1009093.

5.	Thommes M, Wang T, Zhao Q, Paschalidis IC, Segrè D. Designing Metabolic Division of Labor in Microbial Communities. mSystems. 2019;4(2):e00263-18. doi: doi:10.1128/mSystems.00263-18.

6.	García-Jiménez B, García JL, Nogales J. FLYCOP: metabolic modeling-based analysis and engineering microbial communities. Bioinformatics. 2018;34(17):i954-i63. doi: 10.1093/bioinformatics/bty561.

7.	Luo H, Li P, Ji B, Nielsen J. Modeling the metabolic dynamics at the genome-scale by optimized yield analysis. Metabolic Engineering. 2023;75:119-30. doi: <https://doi.org/10.1016/j.ymben.2022.12.001>.
