# Supplementary material for: A structured evaluation of genome-scale constraint-based modeling tools for microbial consortia
Source: PLoS Comput Biol. 2023 Aug 14;19(8):e1011363. doi: 10.1371/journal.pcbi.1011363 (PMC10449394; doi:10.1371/journal.pcbi.1011363)
Supplement: Attachment — Submitted filename: PLOSComp-Rev2-ResponseLetter.docx [file pcbi.1011363.s018.docx]

**Ms. Ref. No.:	PCOMPBIOL-D-23-00221R1-****PLOS Computational Biology**

**Manuscript Title:** A Structured Evaluation of Genome-Scale Constraint-Based Modeling Tools for Microbial Consortia

**Article Type:** Original research paper

Dear Prof. Tunahan Çakır, Ph.D. – Guest Editor of *PLOS Computational Biology* and Prof. Kiran Patil, Ph.D. – Section Editor of *PLOS Computational Biology*,

We would like to thank the reviewers for their insightful comments. Our responses to their comments are listed below (comments and questions from a reviewer are in bold, followed by our responses). The lines indicated in the comments correspond to the new version of the manuscript. We believe we have improved the manuscript with the reviewer’s constructive comments and hope it is ready for publication.

******Reviewer 1******

# GENERAL COMMENT 1

# The authors have satisfactorily answered my previous concerns and the work is more than suitable for publication. I consider that the current manuscript is a valuable piece for the community interested in modeling and engineering microbiomes.

We would like to express our sincere gratitude for your positive feedback and for acknowledging our efforts in addressing your previous concerns. We are thrilled to hear that you find our work suitable for publication and consider it a valuable contribution to the community interested in modeling and engineering microbiomes.

Your affirmation boosts our confidence in the significance of our research and its potential impact. We have worked diligently to ensure the quality and rigor of our study, and it is gratifying to receive such positive validation from an esteemed reviewer like yourself.

We deeply appreciate your time and expertise in evaluating our manuscript. Your favorable assessment motivates us to continue our efforts in advancing the field of genome-scale metabolic modeling of microbial communities. We are committed to sharing our findings with the scientific community and contributing to the collective knowledge in this area.

Once again, thank you for your encouraging words and your endorsement of our work. Your support means a great deal to us, and we look forward to sharing our research with a wider audience.

******Reviewer 2******

# GENERAL COMMENT 1

# The repository authors provided is exceptionally challenging to navigate and understand. There are hundreds of files, this is unclear how to run them to get the figure the authors provide in the manuscript. Many methods are just shown as they are, with documentation from original papers, with tons of comment code. Thus it remains unclear how to reproduce the study (what are the input files for the figures, and how did you generate them?). I highly recommend that authors have another revision and seriously address this comment; this will improve the citation count of their work and provide a valuable resource for the community. There is too much irreproducible research out there, and this work should not be adding to that pile. I am not asking to containerise everything, but having a script showing how to plot individual figures and how the input files for figures were generated is essential (at least commenting on which tool was run).

Thank you for taking the time to review our manuscript and provide your valuable feedback. We appreciate your constructive comments regarding the accessibility and reproducibility of our repository. We apologize for any confusion caused by the large number of files and the lack of clarity on how to run them to obtain the figures presented in the manuscript.

We completely understand your concerns about the difficulty in reproducing our study and the importance of providing clear instructions to facilitate the replication of our results. We agree that reproducibility is a fundamental aspect of scientific research and strive to address this issue effectively.

Based on your feedback, we have revised the repository and implemented measures to enhance its navigability and understandability. Our aim is to provide a valuable resource for the community and improve the citation count of our work.

To address your specific concerns, we included a detailed script that demonstrates how to plot individual figures for each category of tools (see the updated GitLab repository: <https://gitlab.com/wurssb/Modelling/modelingtools_microbial_consortia> as well as the latest version of the Zenodo repository: DOI: 10.5281/zenodo.8074832). These scripts explicitly mention and contain the input files required for generating each quantitative figure, ensuring that researchers can easily replicate our results. Additionally, we provided clear documentation within the repository in the scripts as well as accompanying README files, specifying the tools used and the inputs needed to create the figures.

While we understand your suggestion regarding containerization, we focused on improving the accessibility of the existing Gitlab repository as well as the updated Zenodo repository and ensuring that it is as user-friendly as possible. We also understand there are many files contained within both repositories. However, these files are related to the tool architecture (necessary functions related to running the tool) or outputs generated from our study’s model simulations. Our intention is to strike a balance between providing detailed instructions, reporting our findings, and overwhelming users with unnecessary complexity.

We sincerely appreciate your feedback, as it helped us improve the quality and reproducibility of our research. By addressing your concerns, we hope to provide a more accessible and comprehensible repository that benefits the scientific community. Please rest assured that we take your recommendations seriously and made every effort to implement them effectively.

Thank you once again for your valuable input. We are committed to making the necessary revisions and providing a clear and reproducible resource for the community.

Sincerely,

Dr. William T. Scott, Jr.
